# RCSB Protein Data Bank: Sustaining a living digital data resource that enables breakthroughs in scientific research and biomedical education

**DOI:** 10.1002/pro.3331

**Published:** 2017-11-11

**Authors:** Stephen K. Burley, Helen M. Berman, Cole Christie, Jose M. Duarte, Zukang Feng, John Westbrook, Jasmine Young, Christine Zardecki

**Affiliations:** ^1^ Research Collaboratory for Structural Bioinformatics Protein Data Bank Institute for Quantitative Biomedicine, Rutgers, The State University of New Jersey Piscataway New Jersey 08854; ^2^ Rutgers Cancer Institute of New Jersey, Robert Wood Johnson Medical School New Brunswick New Jersey 08903; ^3^ Research Collaboratory for Structural Bioinformatics Protein Data Bank San Diego Supercomputer Center, University of California, San Diego La Jolla California 92093

**Keywords:** Research Collaboratory for Structure Bioinformatics, RCSB, Protein Data Bank, PDB, Worldwide Protein Data Bank, wwPDB, PDBx/mmCIF, chemical component dictionary, crystallography, NMR spectroscopy, 3D electron microscopy, integrative/hybrid methods, data archive, macromolecular structure, biocuration, validation, metadata, FAIR principles, open access, data deposition

## Abstract

The Protein Data Bank (PDB) is one of two archival resources for experimental data central to biomedical research and education worldwide (the other key Primary Data Archive in biology being the International Nucleotide Sequence Database Collaboration). The PDB currently houses >134,000 atomic level biomolecular structures determined by crystallography, NMR spectroscopy, and 3D electron microscopy. It was established in 1971 as the first open‐access, digital‐data resource in biology, and is managed by the Worldwide Protein Data Bank partnership (wwPDB; wwpdb.org). US PDB operations are conducted by the RCSB Protein Data Bank (RCSB PDB; RCSB.org; Rutgers University and UC San Diego) and funded by NSF, NIH, and DoE. The RCSB PDB serves as the global Archive Keeper for the wwPDB. During calendar 2016, >591 million structure data files were downloaded from the PDB by *Data Consumers* working in every sovereign nation recognized by the United Nations. During this same period, the RCSB PDB processed >5300 new atomic level biomolecular structures plus experimental data and metadata coming into the archive from *Data Depositors* working in the Americas and Oceania. In addition, RCSB PDB served >1 million RCSB.org users worldwide with PDB data integrated with ∼40 external data resources providing rich structural views of fundamental biology, biomedicine, and energy sciences, and >600,000 PDB101.rcsb.org educational website users around the globe. RCSB PDB resources are described in detail together with metrics documenting the impact of access to PDB data on basic and applied research, clinical medicine, education, and the economy.

## Introduction: Protein Data Bank, RCSB PDB, and wwPDB

The Protein Data Bank (PDB) was established with a handful of X‐ray crystal structures of proteins in 1971,^1^ following a landmark Cold Spring Harbor Laboratory Symposium.^2^ It was the first open access digital‐data resource in the biological sciences, and it is no exaggeration to say that the PDB has exemplified the FAIR principles (findability, accessibility, interoperability, and reusability)^3^ since its inception. Today, the PDB is one of two primary archival resources for experimental data that are central to biological and biomedical research and education worldwide (the other key Primary Data Archive in biology being the International Nucleotide Sequence Database Collaboration; insdc.org).

The central role of the PDB and its longstanding global impact derives from the intimate link between form and function that pervades biology. Notwithstanding the 1896 maxim of the visionary American architect Louis Sullivan “life is recognizable in its expression, that form ever follows function,”^4^ form (3D structure) dictates function in biology. Arguably, this relationship became apparent when the discipline of structural biology was born with publication the Watson and Crick double helix model for the structure of DNA.^5^ Reinforced by the ever‐growing wealth of data in the PDB, we appreciate that Sullivan's ideas do not hold for the “architecture” of biological macromolecules. To reiterate, function is determined by 3D structure!

The holdings of the PDB archive also tell us that proteins similar in amino acid sequence encoded by evolutionarily related genes (i.e., similar in nucleic acid sequence) share common 3D structures. These closely related structures typically carry out similar if not identical biological or biochemical functions. Evolution is decidedly parsimonious. Instead of the PDB archive containing a plethora of entirely different 3D structures, the number of distinct spatial configurations of polypeptide chains (i.e., protein domain structures) populating protein fold space is thought to fall somewhere in the range of 3000‐10,000.^6^

Current PDB holdings number 134,436 atomic level structures of proteins, DNA, and RNA experimentally determined by macromolecular X‐ray crystallography (MX: ∼90%), nuclear magnetic resonance spectroscopy (NMR: ∼9%), and 3D electron microscopy (3DEM: ∼1%). Nearly three quarters (∼73%) of PDB structures also include one or more ligands (e.g., enzyme co‐factors and inhibitors, US FDA approved drugs, metals), and ∼8% of PDB structures include one or more carbohydrate components. Virtually all PDB structures were determined with the support of research funding from governments or private philanthropies, and the PDB archive is now widely regarded as a global public good. Replacement value of current PDB archival holdings is conservatively estimated at more than 13 billion US dollars.^7^

The PDB archive is managed jointly by the Worldwide Protein Data Bank partnership (wwPDB; wwpdb.org),^8^ consisting of the RCSB Protein Data Bank,^9^, ^10^ Protein Data Bank Japan (PDBj),^11^ the Protein Data Bank in Europe (PDBe),^12^ and BioMagResBank (BMRB).^13^ The wwPDB organization operates under a formal agreement (wwpdb.org/about/agreement), most recently renewed in 2013. This agreement commits wwPDB partners to standardizing, collecting, validating, annotating, and storing macromolecular structure data as a single global archive for *Data Depositors* and disseminating these data *via* FTP to *Data Consumers*, all at no charge with no restrictions on data usage.

US PDB operations are conducted by the RCSB Protein Data Bank (RCSB PDB; RCSB.org) at Rutgers, The State University of New Jersey and the San Diego Supercomputer Center (University of California San Diego, UCSD). The RCSB PDB also serves as the Archive Keeper, responsible for ensuring disaster recovery of PDB data and coordinating weekly archival updates among wwPDB partners.

The vision of the RCSB PDB is “To enable breakthroughs in scientific inquiry, medicine, drug discovery, technology, and education with rich structural views of biological systems.”

The mission of the RCSB PDB is as follows:

1. Enabling efficient deposition, high‐quality curation, and exploration of data in the global PDB archive.
2. Leading biological structure representation and driving integration with related data resources.
3. Establishing and fostering communication and collaboration across sciences.
4. Inspiring, enabling, and informing diverse users through structural views of biology and medicine.

Central responsibilities include Biocuration, Archive Management, and Data Delivery, all supported by robust infrastructure that ensures 24/7/365 support of PDB *Data Depositors* and *Data Consumers* worldwide. In addition to managing day‐to‐day operations, the RCSB PDB must address the challenge of sustaining the PDB as a living data resource in the face of relentless growth in the number and complexity of depositions, disruptive changes in information technology, scientific and technical advances in structure determination made by our *Data Depositors*, and the evolving needs of our growing *Data Consumer* community.

## Week in the Life of the RCSB PDB

**Figure 1:**
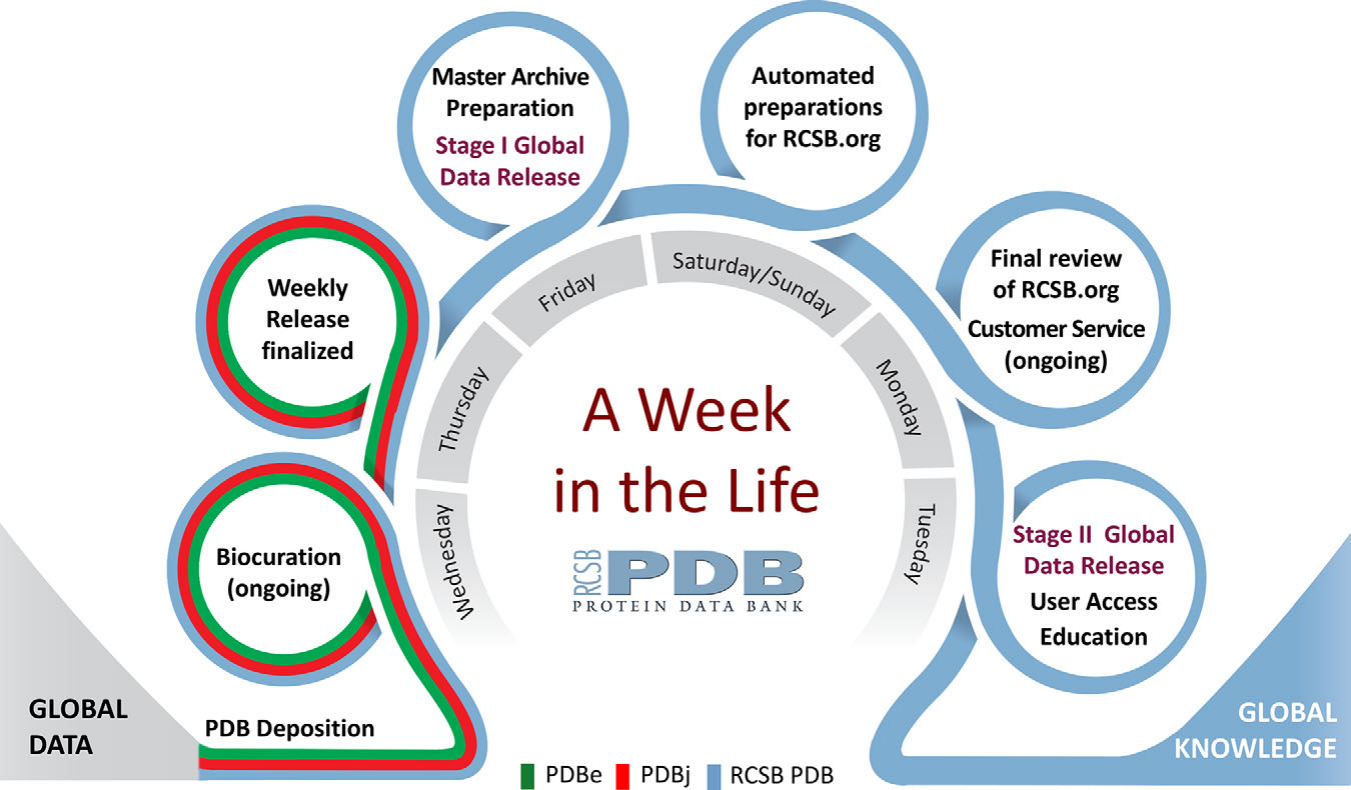
Week in the life of the RCSB PDB, showing the progression from data deposition at wwPDB regional data centers to preparation and finalization of weekly releases by the RCSB PDB acting as PDB Archive Keeper, followed by Stage I (partial) and Stage II (full) Global PDB Data Release.

Every week of the year, the RCSB PDB, PDBe, and PDBj together receive ∼250 new structures from *Data Depositors* working on every inhabited continent. [RCSB PDB receives data from the Americas and Oceania (∼45%); PDBe from Europe and Africa (∼35%); and PDBj from Asia and the Middle East (∼20%)]. The weekly cycle depicted in Figure 1, repeated 52 weeks a year, begins every Wednesday at 00:01 Universal Time (UTC). Every day of the year, incoming data are validated, biocurated, and packaged for public release at each of the wwPDB regional data centers in US, Europe, and Asia. Every Thursday, structures scheduled for release from PDBe and PDBj in that particular week are transmitted to RCSB PDB. Acting as PDB Archive Keeper, RCSB PDB performs final checks for consistency and readies the data for public release. Every Friday, 200 plus new structures are added to the PDB Master Archive, which is stored on the wwPDB FTP site (ftp.wwpdb.org) and replicated on redundant FTP sites maintained by RCSB PDB (ftp.rcsb.org), PDBe (ftp.ebi.ac.uk/pub/databases/pdb/), and PDBj (ftp.pdbj.org).

Every Saturday at 03:00 UTC, Stage I Global PDB Data Release (partial) makes the following data publicly available for each new PDB entry: PDB ID, protein and nucleic acid sequences of each polymer, crystallization pH, and ligand SMILES strings. Release of these data supports blind challenges involving prediction of 3D protein structure [CAMEO: cameo3d.org^14^ and ligand binding pose D3R CELPP: drugdesigndata.org/about/celpp]. These blind challenges enable automated methods development in bioinformatics and computational chemistry/drug discovery, respectively.

Preparations for Stage II Global PDB Data Release (full) by the RCSB PDB involve the following steps. Every weekend, data from ∼40 external biological data resources are automatically integrated with each new PDB entry scheduled for public release *via* the RCSB.org website. Every Monday, the updated version of RCSB.org undergoes final quality review. Every Tuesday, the updated version of RCSB.org is readied for public release [with the PDB‐101 educational website (pdb101.rcsb.org) and the wwPDB website (wwpdb.org) updated as needed]. Every Wednesday at 00:00 UTC, the updated PDB archive is made publicly available *via* the wwPDB FTP site and the wwPDB partner FTP sites. Shortly thereafter, power users of the PDB archive as a whole download all of the newly released structures for in‐house use by academic computational biologists and chemists, pharmaceutical and biotechnology companies, and >200 biological data resources that repackage and distribute PDB. Every Wednesday at 00:00 UTC, the updated PDB archive is made available to *Data Consumers* around the world *via* RCSB.org and wwPDB partner websites.

## Deposition/Validation/Biocuration of New PDB Structures from *Data Depositors*

Since 2014, PDB data have been stored and distributed using the PDBx/mmCIF master format,^15^ which is managed by the wwPDB partners in collaboration with expert community stakeholders serving on the PDBx/mmCIF Working Group (wwpdb.org/task/mmcif). At the time of writing, the PDBx/mmCIF dictionary contained more than 4400 data items, including ∼250 and ∼1200 specific to NMR and 3DEM, respectively. The PDBx/mmCIF dictionary and PDBx/mmCIF format data files are fully machine‐readable, and no domain knowledge is required to read the files.

**Figure 2:**
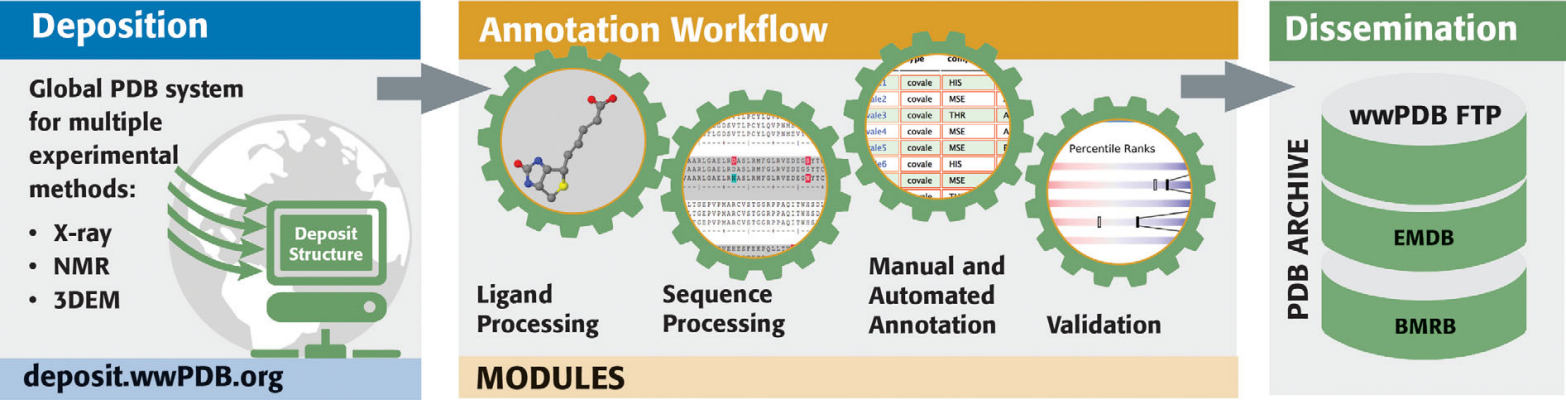
OneDep system workflow.

Worldwide Protein Data Bank partners use the OneDep system for deposition, validation, and biocuration of MX, NMR, and 3DEM structures (Fig. 2) coming into the PDB archive on a daily basis.^16^ Within this unified global system, RCSB PDB operates independent OneDep access sites from both Rutgers and UCSD. Bicoastal redundancy ensures that PDB Data Depositors in the Americas and Oceania have continuous access to the OneDep system. In parallel, PDBe operates a OneDep access site in the UK (serving Europe and Africa), and PDBj operates a OneDep access site in Japan (Asia and the Middle East). In the event that OneDep access sites located outside the US become inoperable, depositions are redirected to ensure uninterrupted access for *Data Depositors* worldwide.

PDB data depositions contain comprehensive descriptions of structural models coming from MX, NMR, and 3DEM. Each archival entry is denoted by a 4‐character PDB identifier (e.g., 1vtl). In addition to atomic coordinates, details regarding the chemistry of biopolymers and any bound small molecules are archived, as are metadata describing biopolymer sequence, sample composition and preparation, experimental procedures, data‐processing methods/software/statistics, structure determination/refinement procedures and statistics, and certain structural features (e.g., secondary and quaternary structure). Experimental data coming from MX (represented as structure factor amplitudes or intensities) and NMR (restraints and chemical shifts) must be archived in the PDB. Mass density maps from 3DEM must be archived in the EMDB (https://www.ebi.ac.uk/pdbe/emdb/), also submitted *via* the OneDep system. Deposition of atomic coordinates, experimental data, and metadata are required by all major scientific journals when publishing a new structure determination study.

**Figure 3:**
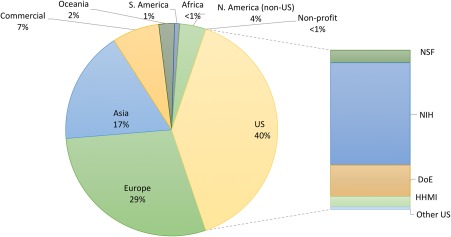
PDB depositions by geography (January 2000‐June 2017) and breakdown of funding source for US depositions.

Geographic breakdown of PDB data coming from *Data Depositors* working on every inhabited continent is provided in Figure 3. In 2016, total depositions numbered 11,614 (MX: ∼91%; NMR: ∼4%; 3DEM: ∼5%). Annual data deposition statistics 2000–2017 can be found on the wwPDB website (http://www.wwpdb.org/stats/deposition). Ph.D.‐trained biocurators at RCSB PDB processed 5323 incoming depositions during calendar 2016, providing annotation and validation services to ∼3500 *Data Depositors* based in the Americas and Oceania. Expert biocuration of incoming structures and related experimental data and metadata is controlled by the OneDep system workflow manager (developed jointly by RCSB PDB, PDBe, PDBj, and BMRB), which ensures that new PDB structures conform to a common global standard.

Since 2014, structures coming into the PDB archive have been validated by the OneDep system using processes developed jointly by the wwPDB and expert, method‐specific Validation Task Forces.^17^, ^18^, ^19^ An official wwPDB Validation Report is produced for every new PDB structure when the *Data Depositor* finalizes the entry for public release. Increasingly, scientific journals are requiring submission of these reports together with the manuscript describing the structure determination study. wwPDB Validation Reports are made available on the RCSB.org website for every structure at the time of public release. On an annual basis, these reports are re‐calculated across the PDB archive (with reference to updated structure quality statistics for the entire corpus of data). RCSB PDB recently published a “before *versus* after” assessment of the impact of the wwPDB Validation Report,^20^ documenting improvement in quality for macromolecule structures determined by MX. The wwPDB also operates a stand‐alone validation server (validate.wwpdb.org), which produces an unofficial wwPDB Validation Report intended for use during structure determination and refinement. wwPDB provides Validation Report documentation and User Guides at http://www.wwpdb.org/validation/validation-reports/.

Introduction of the OneDep system in 2014 has benefited wwPDB biocurators and PDB *Data Depositors* alike. Biocurators are now processing incoming structures using an internationally‐agreed set of common standards that promise to improve both PDB data quality and consistency going forward. The OneDep system has also improved the efficiency of biocuration, and has for the first time enabled facile remediation of data files across the entire archive. (For example, the archive was recently upgraded to V5.0 of the PDBx/mmCIF dictionary with significantly less effort that earlier, less extensive dictionary version upgrades.) *Data Depositors* are benefiting from more stringent validation and biocuration than was previously possible with the multiple legacy systems in use prior to 2014. Of particular significance has been the impact of the wwPDB Validation Report. Since its introduction, approximately 25% of new depositions undergo major corrections by *Data Depositors* as a consequence of the combined feedback from expert biocurators and the wwPDB Validation Report. It is hoped that this metric will decline going forward now that the stand‐alone wwPDB validation server is available for use prior to deposition using the OneDep system.

Finally, the OneDep web user interface provides for secure transfer of *Data Depositors*’ confidential information. Each deposition session is password protected and a secure web protocol (HTTP over TLS/SSL) is used. Prior to public data release into the PDB archive, deposition session data access is limited to the *Data Depositors* and wwPDB biocurators. Communications regarding the content of a given PDB deposition are conducted through the password‐protected secure web channel.

## Safeguarding, Releasing, and Remediating PDB Archival Contents

At the beginning of 2017, the PDB archive contained 125,463 entries and is expected to grow by ∼10% before year end. Total data storage for the archival files currently totals >750 GB. Total data storage for archival files plus related records, and so forth, currently totals ∼12 TB.

RCSB Protein Data Bank and its other wwPDB partners maintain synchronized copies of the FTP repository. The RSYNC protocol used for data synchronization provides an internal checksum mechanism as part of the data transfer operation to ensure consistency among FTP repository copies. Additional indices of content are provided to allow software tools at each site to further verify the completeness and correctness of updates as they occur.

As Archive Keeper, RCSB PDB releases of data on a coordinated weekly schedule. Data files are rechecked before delivery into the repository. RCSB PDB coordinates periodic remediation of the archive by wwPDB members. These exercises range in complexity from making identical updates to small numbers of PDB structures (correcting errors of common origin) to changes across the entire archive, such as when the PDBx/mmCIF dictionary is updated (https://www.wwpdb.org/documentation/remediation).

Finally, as PDB Archive Keeper, the RCSB PDB is responsible for safe and secure storage of all archival files plus related records. These data are stored on multiple RCSB PDB servers located at Rutgers, UCSD, and a third undisclosed, secure location. At the end of each year and at milestone archive update, the RCSB PDB records “snapshots” of the PDB archive. These snapshots are made available for FTP download from multiple sites (wwPDB/RCSB PDB: ftp://snapshots.wwpdb.org/; PDBj: ftp://snapshots.pdbj.org/).

## Disseminating the PDB Archive to *Data Consumers*

Structural data flow out of the PDB archive to *Data Consumers* working in every sovereign nation recognized by the United Nations. During calendar 2016, structure data file downloads totaled >591 million or ∼1.5 million/day. The sources of these downloads were as follows: Global FTP structure data file downloads ∼366 million, plus RCSB.org ∼161 million, PDBe website ∼44 million, and PDBj website ∼20 million). Structure data file download statistics 2000–2017 are provided on the wwPDB website (http://www.wwpdb.org/stats/download).

RCSB Protein Data Bank web services further expose a rich collection of tools enabling programmatic access to primary and value‐added data, search and reporting services, and comparative sequence and structure analysis tools. The growth rates for these programmatic service offerings are among the highest for all RCSB PDB services.

RCSB Protein Data Bank also provides tools to support efficient mirroring of the repository data. Rsync services accounted for 110 million data file downloads in the first half of 2017. These services support users maintaining synchronized copies of the PDB archive, such as private company databases, computational chemistry resources, and other life science web resources. Surveying articles in the annual Database Issue of *Nucleic Acid Research* (http://www.oxfordjournals.org/nar/database/a/), we identified >200 data resources reporting a dependency on RCSB PDB data and services.

**Figure 4:**
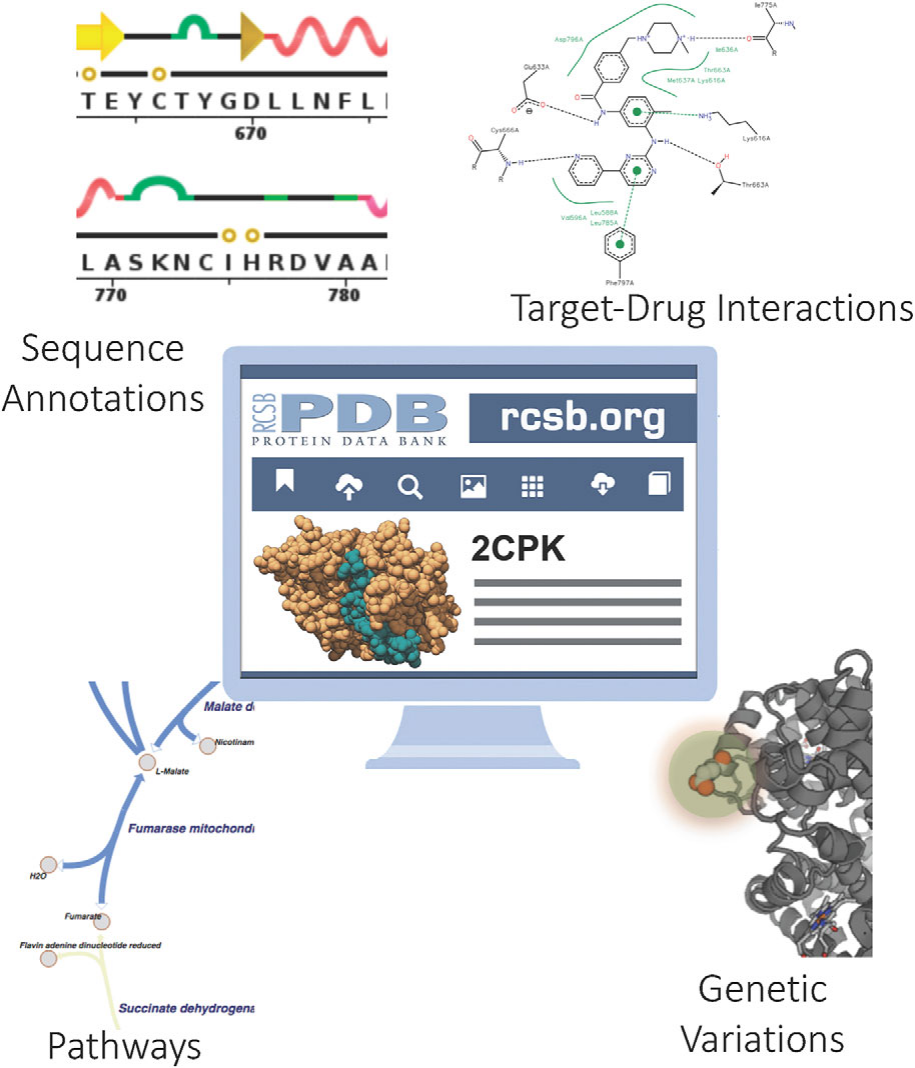
Data integrated from external resources enables research. Information about publications, sequence annotations, drug interactions, and more are updated regularly to enable *Data Consumers* to browse entire PDB archive by external annotations, access annotations for individual structures, and visualize data in 2D and 3D. Examples shown, clockwise from the upper right: Images from PoseView^32^ are available on Structure Summary pages; the Gene View tool illustrates correspondences between the human genome and PDB structure; metabolic pathways maps in the Pathway View identify pathway components with PDB structures and homology models.^10^

We estimate conservatively that more than 1 million *Data Consumers* worldwide visited RCSB.org during calendar 2016 (∼25% of usage from US). Much of the usage of our website goes well beyond simple download of structure data files. Google Analytics documented an average session duration of ∼6 minutes and a low bounce rate. Our *Data Consumers* utilize RCSB PDB resources (Table 1) online to explore individual structures and groups of structures. Search and reporting tools include PDB data integrated with ∼40 external biological data resources relating to publications in the scientific literature, biochemical and biological function, gene sequence, post‐translational modifications, biochemical pathways, ligand interactions, diffraction data, and so forth (Table 1, Fig. 4). Individual PDB structures are also linked to our PDB‐101 educational website (pdb101.rcsb.org), which was accessed by >600,000 users worldwide in 2016.

**Table 1:** Selected RCSB PDB Resources

Resources to search the PDB archive	Simple top bar search: search by PDB ID, author name, keyword, sequence, or ligand. Autosuggestions are automatically displayed, for example, a query of “protease” launches a suggestion box that organizes possible related UniProt molecule name, structural domain, Enzyme Classification, and so forth
	Pressing return or the search button will perform a plain text search of PDB data, RCSB PDB news, and Molecule of the Month articles
	Advanced Search: Combine multiple searches of specific types of data in a logical AND or OR (e.g., structures with UniProt molecule name “HIV‐1 Protease” with resolution less than 1.5 Å)
	Chemical Component Searching: search for ligand descriptions or structures containing particular small molecules by ID, InChI descriptor, formula, or chemical structure
	Annotation browsers: provide access to structures in the PDB archive using different hierarchical classification trees, including the Anatomical Therapeutic Chemical (ATC) Classification System from the WHO Collaborating Centre for Drug Statistics Methodology; Membrane Proteins identified using the mpstruc database,^21^ sequence clustering, and data derived from UniProt^22^; Protein Symmetry; Biological Process, Cellular Component, and Molecular Function as annotated by Gene Ontology (GO)^23^; Enzyme Classification; Transporter Classification; Source Organism (NCBI); Genome Location (NCBI); MeSH (Medical Subject Headings) from NCBI^24^; SCOP description of evolutionary and functional relationships^25^; and CATH^26^ clustering of proteins at four major levels
	Sequence searching: find structures matching or similar to a given sequence by either entering a target sequence or using a particular chain in a PDB structure
Search results	To explore individual entries returned from a search, Structure Summary pages provide an overview key information about an entry with options to download data, search for similar structures containing the same data (e.g., classification, author, organism, ligand), and options to view in 3D, explore external annotations, study the sequence, compare the sequence and 3D structure with other PDB structures, and access information about the experiment
	Query results containing multiple entries can be further refined (by organism, molecule name, taxonomy, etc.), sorted (by size, release date, resolution, etc.), or explored by individual entry. A variety of tabular reports can be generated (described below)
Resources for visualization	Structure summary pages off links to interactive 3D views in NGL^27^ and other viewers that enable different ways to view an entry Special visualization features include visualization of the ligand interactions in 2D and 3D, 3D view of the binding pocket, and 3D display of the molecule and electron density
	Protein Feature View is a graphical summary of a full‐length protein sequence from UniProt and its correspondence to PDB entries, annotations from external databases (such as Pfam), homology models information from the Protein Model Portal, predicted regions of protein disorder, and hydrophobic regions
	Human Gene View is a tool for visualizing correspondences between the human genome^28^ and PDB structure
	Pathway View maps metabolic pathway components^29^ with PDB structures and homology models
Resources for analysis	Tabular reports can be generated for a set of structures that can be customized to include ∼150 data items. Summary reports can also be generated
	Comparison Tool^30^ uses several algorithms to calculate pairwise sequence and structure alignments Comparisons can be made for any protein in the PDB archive and for customized or local files not in the PDB. Special features include support for both rigid‐body and flexible alignments and detection of circular permutations^31^

## Impact of the PDB Archive and the RCSB PDB

Research and education communities have benefited enormously from open access to the PDB digital data resource since 1971. The discipline has grown from a few hundred *Data Depositors* in the 1980s to >30,000 today, while their collective work product (i.e., the contents of the PDB archive) has grown from fewer than 100 PDB structures in 1980 to more than 130,000 today. The archive is growing at the rate of ∼10% per year, which corresponds to a doubling time of ∼6 years. Structures represented in the PDB archive come from every kingdom of life, and provide important insights into every area of biology and medicine. The PDB is among the most heavily used digital data resources in basic and applied biomedical research and education. In a 2015 study, the PDB archive was identified as second only to clinicaltrials.gov in biological data resource usage by NIH‐funded researchers.^33^

Open access to PDB data has both accelerated the development of structural biology as a discipline and promoted a robust culture of reproducibility. Within the sub‐discipline of MX, most structures (∼72% in 2016) are now determined by molecular replacement^34^ using one or more structures from the PDB archive to overcome the Phase Problem. Development of macromolecular NMR and 3DEM as mainstream methods in structural biology benefited significantly from availability of data from the PDB archive, BioMagResBank, and EMDB. Data sharing and the value added by wwPDB biocurators and various structure validation systems has also contributed to reproducibility of all three experimental methods represented in the PDB archive.

The availability of the corpus of data in the PDB has made it possible to do analyses of groups of structures in order to understand common principles. Without the PDB archive, it would not have been possible to establish realistic validation criteria for individual structures. The PDB archive has also been used to classify and understand protein domains. There are resources that provide these classifications and group them into Superfamilies.^6^, ^25^ Protein‐protein interactions^35^ and protein‐nucleic interactions have also been analyzed across the PDB archive.^36^ More than 1000 papers describing these types of analyses and resources have been published.^37^ Indeed, the entire field of structural bioinformatics has been enabled by the existence of the PDB.^38^

The impact of individual structures in the PDB archive can be evaluated by considering the publications describing determination of one or more structures. An RCSB PDB study completed in June 2017 using the Web of Science (wokinfo.com) documented that 109,227 PDB structures have been reported in 52,296 publications. These publications were in turn cited by >2.6 million publications, covering subject areas ranging from Agriculture to Zoology, with 37,749 of the 52,296 published PDB primary citations having been cited 10 or more times (i.e., i10‐index = 37,749).

**Figure 5:**
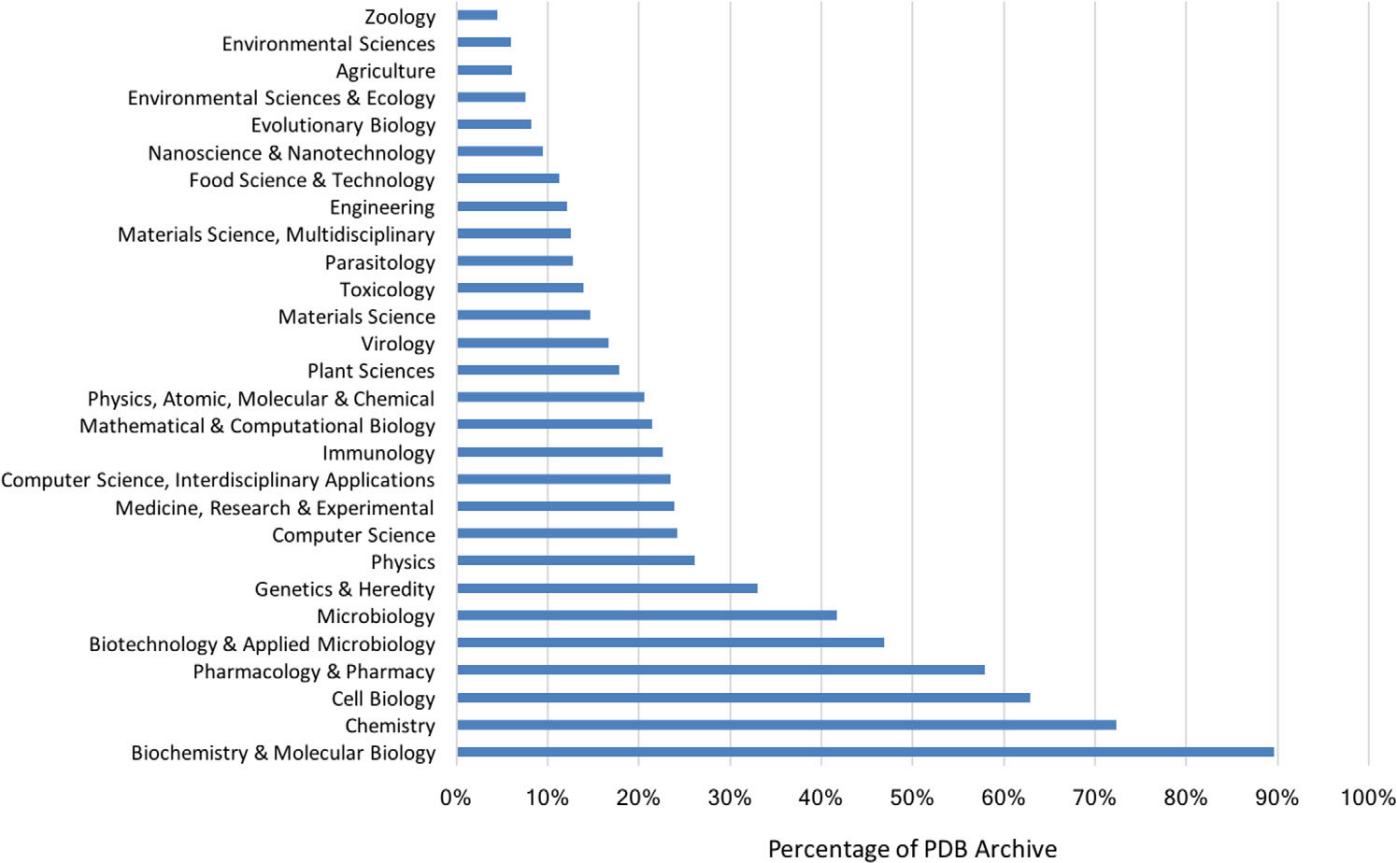
Fraction of published PDB structures cited in subject‐area publications. The impact of individual structures can also be assessed using PDB archive data download statistics. An RCSB PDB study completed in July 2017 documented that each PDB structure has been downloaded an average of ∼30,400 times since 2007. Some PDB structures are extremely “popular.” The top 1% of downloaded structures have each been downloaded an average ∼105,000 times since 2007. Individual structure download statistics are provided on the wwPDB website (www.wwpdb.org/stats/search).

Of equal significance is the question of impact of the PDB archive across the sciences. Our Web of Science analyses permitted estimation of the fraction of the 109,227 published PDB structures cited by publications from different disciplines (Fig. 5). Not surprisingly, nearly 90% of published PDB structures have been cited by journals in the area of Biochemistry & Molecular Biology. High impact within other areas of biomedicine (*Cell Biology*, *Pharmacology and Pharmacy*, *Microbiology*, *Genetics & Heredity*) was also expected. The breadth of the impact of the PDB archive across the sciences came as a surprise, albeit a pleasant one. For example, Computer Science and Physics journals have cited ∼25% of published PDB structures. Equally gratifying is the impact of the PDB archive in subject areas such as Materials Science (∼15%) and Engineering (∼12%), and Evolutionary Biology (∼8%), Environmental Sciences (∼6%), Agriculture (∼6%), and Zoology (∼4%).

**Figure 6:**
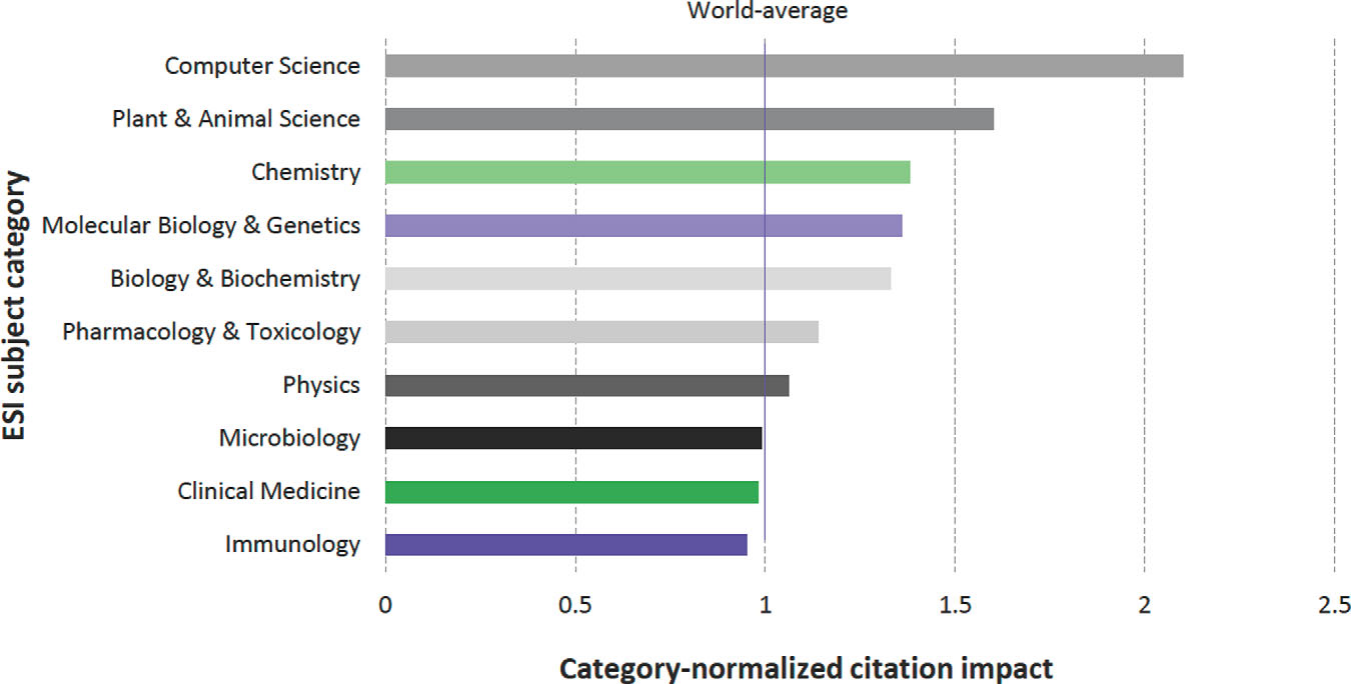
Category‐normalized citation impact of publications in different subject categories citing Berman et al. (2000) from Clarivate Analytics.^37^

The primary RCSB PDB reference, Berman et al.^9^ is highly cited, but typically not by users of individual PDB structures. Hence, citations of the reference provide a means of assessing the impact of the PDB archive as a whole. In 2014, this article was ranked 92nd in the top 100 all time cited publications by the Web of Science.^39^ A recent Clarivate Analytics study commissioned by the RCSB PDB and concluded in May 2017 demonstrated the breadth of impact of the PDB archive across the sciences.^37^ Of particular note from this work was the opportunity to consider the impact of Berman et al. (2000) after normalization for discipline‐dependent citation practices. Figure 6 documents that the PDB archive has an above World‐average impact in the Essential Subject Indicator (ESI) subject categories of Computer Science, Plant & Animal Science, Chemistry, Molecular Biology & Genetics, Biochemistry & Molecular Biology, and Pharmacology & Toxicology.

Literature citations tell only part of the story. In 2015, the RCSB PDB published the outcome of an analysis of PubMed Central holdings^40^ that documented rapid growth of mentions of RCSB.org (2005–2013), largely at the expense of citing Berman et al.^9^ Independent literature reviews conducted by the RCSB PDB revealed frequent mentions of PDB IDs in the body of the text, again largely at the expense of citing either Berman et al.^9^ or the publication reporting the structure determination. Finally, as of June 2017 ∼22% of the structures in the PDB archive were not associated with any publication (either because the structure was never published or because the publication had not yet appeared in the literature). It is possible to cite these “unpublished” structures using the standard format Digital Object Identifier (DOI) attributed to each PDB structure by the wwPDB OneDep system (e.g., 10.2210/pdb1kip/pdb for PDB ID 1kip).

Having documented that both individual PDB structures and the PDB archive as a whole impact the sciences broadly, going well beyond biomedicine, we now consider the impact the PDB archive and the RCSB PDB in three subject areas Fundamental Biology, Biomedicine, and Energy Research. The value to our *Data Consumers* derives from synergistic combination of Open Access to expertly‐curated PDB archive data and a wide array of RCSB PDB services that contribute to rapid advances in basic and applied research in biology and medicine. No matter how great the immediate excitement, each newly deposited structure represents just the latest “passage” in the 3D book of life. By their very nature, individual structures can tell only part of a biomedical story. RCSB PDB and its wwPDB partners validate and expertly biocurate each new structure coming into the PDB archive from our *Data Depositors*. The RCSB PDB website (RCSB.org) systematizes and integrates structural information with ∼40 external data resources for our *Data Consumers*. Collections of individual PDB structures can then be assembled into coherent 3D “stories” with the power to explain entire areas of biology or medicine.

**Figure 7:**
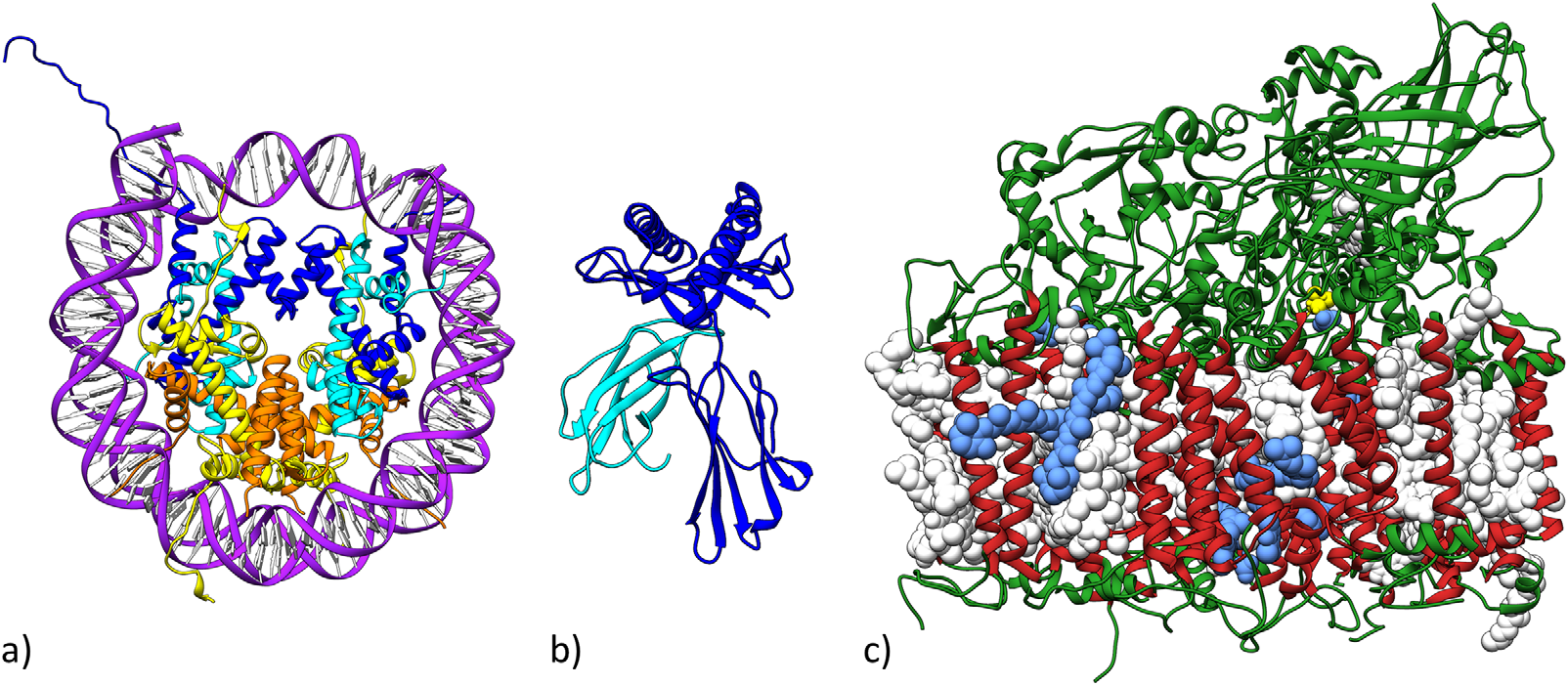
Representative PDB structures that exemplifying impact on our understanding of Fundamental Biology, Biomedicine, and Energy Research. (a) Nucleosome Core Particle (PDB ID 1aoi^41^); (b) Major Histocompatibility Complex 1 (1hla^43^); (c) Photosystem II (1s5l^47^).

Figure 7 illustrates three landmark structures that each served as “index cases” for structure determination campaigns undertaken by PDB *Data Depositors* that in turn transformed our understanding of important areas of science.

The structure of the Nucleosome Core Particle [PDB ID 1aoi^41^] published in 1997 [Fig. 7(a)] is an exemplar of Fundamental Biology research that accelerated efforts to understand in 3D how the DNA genomes of eukaryotes are reversibly packaged into compact chromosomes. The Nucleosome Core Particle structure is the most cited structure in the PDB archive, garnering >4600 citations and >250,000 downloads (since 2007) as of June 2017. More than 300 related structures now available from the PDB archive together help explain DNA packaging at the molecular level [reviewed in part in Ref. 42]. Driven by these structures and insights therefrom, we now appreciate that some of the enzymes catalyzing chemical modification of histone proteins represent potential targets for anti‐cancer drug discovery. Structure‐guided drug discovery efforts in this arena within pharmaceutical and biotechnology companies worldwide rely heavily on Open Access to PDB data with no limitations on data usage.

The structure of Major Histocompatibility Complex 1 [MHC1; PDB ID 1hla^43^] published in 1987 [Fig. 7(b)] is an exemplar of Biomedical Research that launched an effort to understand in 3D how antigens are presented to T‐cells and eventually how T‐cell killing of cancer cells is regulated. The MHC1 structure is the fourth most cited structure in the PDB archive, garnering >3000 citations and >60,000 downloads (since 2007) as of June 2017. More than 750 related structures now available from the PDB archive have together transformed our understanding of T‐cell biology [reviewed in part in Refs. 44, 45, 46]. Before these structures were determined and made publicly available, our knowledge of T‐cell function and regulation was woefully inadequate. These structures unambiguously defined the mechanisms by which the human immune system recognizes abnormal cells arising from malignancy and infection, and catalyzed efforts to develop strategies for directed modulation the immune response for therapeutic purposes. The recent revolution in immune checkpoint anti‐cancer therapies that contributed decisively to President Jimmy Carter's unexpected recovery from end‐stage metastatic melanoma following treatment with the Merck monoclonal anti‐PD1 antibody (Keytruda) can be traced back to the 1987 structure of MHC1.

The structure of Photosystem II [PS_II; PDB ID 1s5l^47^] published in 1985 [Fig. 7(c)] is an exemplar of Energy Research that initiated efforts to understand in 3D how photosynthetic molecules harness the energy of sunlight and feed the world. The PS_II structure is the 13th most cited structure in the PDB archive, garnering ∼2200 citations and >100,000 downloads (since 2007) as of June 2017. More than 400 structures now available from the PDB archive help explain how PS_II, Cytochrome b_6_f, Plastocyanin, and Photosystem I and other molecular actors work together to convert light energy into chemical energy in plants [reviewed in part in Refs. 48, 49]. Collectively, these structures are informing research into renewable energy production *via* bio‐inspired catalysts and sustainable biofuels.

In addition to developing these compelling, albeit anecdotal, reports of impact, the RCSB PDB recently undertook a more quantitative assessment of the value of US‐funded PDB structures and the RCSB PDB for our federal funders (NSF, NIH, and DoE). US funding acknowledgements in publications reporting PDB structures available from the Web of Science were analyzed to generate Table 2. At the time of writing, PDB *Data Depositors* had acknowledged US funding of >63,000 structures (NSF ∼7%; NIH ∼75%; DoE ∼18%), every one of which was biocurated and validated by the RCSB PDB. These US‐funded structures are described in >27,000 publications (NSF ∼7%; NIH ∼77%; DoE ∼16%). Collectively, these publications have been cited >1 million times, giving an average number of citations per paper of ∼40.

**Table 2:** Impact of US‐Funded PDB Structures as Measured by Number of Publications and Citations Thereof, with Fraction Represented Within the Top 40% of Global PDB Downloads (Since 2007)

	Number PDB depositions funded	Publications	Citations	% in Top 40% of PDB downloads
NSF‐funded	4577	1841	56,026	97%
NIH‐funded	47,347	21,340	874,367	98%
DoE‐funded	11,201	4406	104,493	98%
**Total**	**63,125**	**27,587**	**1,034,886**	**98%**

## Impact on Education and Outreach

Access to PDB data reveal biology in 3D in atomic detail. These structures are central to biomedical education, as evidenced by their increased usage in mainstream textbooks. The 1984 edition of *Fundamental Immunology* did not reference or include any images of structures. By 2013, 57 images and 277 PDB structure citations were included in the 7th edition.^50^ Similarly, *Fundamentals of Biochemistry* has increased the number of PDB images and structures over the years, and began including an “Introduction to PDB” chapter in 2008 that continues to this day.^51^

RCSB PDB hosts an online portal (pdb101.rcsb.org) designed for teachers, students, and the curious public that enables understanding of all aspects of fundamental biology, biomedicine, and energy. Learning materials include on‐line publications, videos animations, posters, templates to build molecular paper models, and curricular materials. These resources are built around our heavily used Molecule of the Month series, which describes the structure and function of macromolecules from Actin to Zika.^52^ In 2017, the PDB‐101 website was highlighted as “Best of the Web” by *Genetic Engineering & Biotechnology News*.^53^ Illustrations created for Molecule of the Month and PDB‐101 have been recognized with awards from the Wellcome Trust, FASEB BioArt awards, and the NSF‐Popular Science Vizzies (http://pdb101.rcsb.org/learn/resources/posters_flyers_and_calendars).

Irving Geis (1908–1997) was a pioneering artist who helped illuminate the field of structural biology with his iconic images of myoglobin, hemoglobin, DNA, and other important biomolecules. Through a collaboration with the Howard Hughes Medical Institute (HHMI), which now owns the Geis Archives, RCSB PDB has established a digital archive of Geis’ molecular art. This resource displays many of Geis’ illustrations in the context of the corresponding PDB structures and related molecular information. Just as Geis’ groundbreaking illustrations have inspired generations of structural biologists, we hope that PDB‐101 materials will serve to inspire generations to come.

## Impact on Private Sector Research and Development

PDB archival data and RCSB PDB services are central to research and development in private sector organizations. Virtually every pharmaceutical and biotechnology company drug discovery team is a PDB *Data Consumer*.

Most larger companies maintain copies of the entire PDB archive (downloaded from a PDB FTP site) inside their organization's firewall. Smaller companies use PDB FTP sites, RCSB PDB web services and Rsync, and our RCSB.org website to access PDB data directly. Structure‐guided drug discovery combined with the wealth of Open Access data available (without limitations on usage) from the PDB archive have helped transform HIV/AIDS from a death sentence to a chronic disease that can be managed with combination anti‐retroviral therapy.^54^ For example, HIV Protease Inhibitor discoveries over the years have depended critically on Open Access to >1000 HIV protease crystal structures and ∼3000 other protease structures, which represent possible off‐targets to be avoided during drug discovery. Similar stories abound in the area of structure‐guided discovery of anti‐cancer agents, particularly those targeting protein kinases.

Going beyond these anecdotes, there is quantitative evidence that PDB data are contributing to patent application filings by both academic and industrial inventors. Directed searches by RCSB PDB in June 2017 for PDB mentions using the US Patent and Trademark Office website (uspto.gov) identified ∼20,000 in‐process patent applications and ∼6500 issued US patents that included PDB mentions. Analogous searches of the global patent literature using patseer.com documented >50,000 issued patents and patent applications in process worldwide that include PDB mentions.

## Impact on the Economy

The RCSB PDB has also used the wealth of available data regarding RCSB.org utilization to try to quantify the economic impact of the resource. A study by the Rutgers Office of Research Analytics (ORA; ora.rutgers.edu) documented various contributions to the local, national, and global economies.^7^ The most significant of these contributions concerns the so‐called Use Value of the RCSB.org website. Widely‐accepted economic theory holds that the minimum value that can be attributed to any activity is the actual costs borne by the individual engaged in that activity. As RCSB.org is Open Access, the most readily quantifiable cost to the individual (or their employer) is the salary plus fringe benefits monies paid to the individual while using the website and PDB data offline. By combining comprehensive Google Analytics data on RCSB.org usage, including geographic location and time spent using the website, with published average salary information for workers in each geography the Rutgers ORA report estimated the Use Value of the website alone to be ∼US$5.5 billion per annum. Federal funding of the RCSB PDB, therefore, provides an excellent Return on Investment for the NSF, NIH, and DoE and ultimately US taxpayers!

The foregoing analysis does not include any attempt to estimate economic impact of PDB data on the societal benefits coming from pharmaceutical and biotechnology companies. While quantitative estimates of the impact of Open Access to PDB data by the private sector are not feasible, information gleaned from public US Securities and Exchange Commission filings serve to shed some light on the topic. For example, global sales of the top 10 anti‐HIV drugs (many of which benefited from structure‐guided drug discovery approaches) totaled ∼US$18.3 billion in calendar 2015.^55^ Similarly, worldwide sales of two second generation drugs used to treat chronic myeloid leukemia (Sprycel and Tasigna, which can be found bound to their target Abl protein kinase in PDB IDs 2gqg^56^ and 3cs9,^57^ respectively) totaled ∼US$3.25 billion in calendar 2015.^55^ As with the HIV‐protease case study described earlier, discovery of protein kinase inhibitor drugs in both academe and industry depends critically on Open Access to ∼3200 PDB structures of protein kinases that help address the challenge of avoiding binding to unwanted off targets (the cause of side effects) across this very large protein family.

## Overarching Value

Looking beyond these metrics and anecdotes, the overarching value of the PDB can be readily appreciated by considering what would happen if the archive ceased operations one day. It is no exaggeration to say that large fractions of global biomedical research, biotechnology and pharmaceutical company research and development, and biomedical education would begin to collapse. Total loss of PDB data would be catastrophic and likely irreversible, as the replacement cost of the archival contents is estimated conservatively at more than $13 billion US dollars.

The PDB is a primary data archive central to biomedicine worldwide, on par with the International Nucleotide Sequence Database Collaboration responsible for nucleic acid sequence data. Both have been classified by the European ELIXIR partnership as Core Data Resources that are “absolutely critical for the integrity and advancement of life science research,” adding that “If for any reason we were to lose access to these Core Data Resources, it would have a devastating effect not only on science, but also on medicine, industry, and innovation.”^58^

In no small part, the success of the PDB archive derives from the quality of the data, and consequently the trust that scientific and educational communities place in the resource. PDB *Data Consumers* are confident that the atomic coordinate data in the archive are faithful representations of physical reality. Their well‐placed confidence reflects the benefits of long‐standing commitments to expert biocuration,^59^ validation,^60^ and periodic remediation of PDB archival holdings by wwPDB partners.^61^, ^62^

## Challenges Ahead

**Figure 8:**
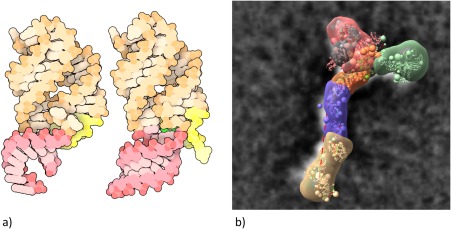
(a) XFEL serial crystallography reveals what happens when adenine binds to a riboswitch^66^ and (b) I/HM multi‐scale structural model of the nuclear pore Nup84 complex.^67^

Beyond managing day‐to‐day operations, the successes of structural biologists generating PDB data and biomedical researchers using PDB data create significant challenges for the RCSB PDB. The needs of our growing user community are continuously evolving. PDB *Data Depositors* are embracing new experimental methods going beyond MX, NMR, and 3DEM, including cryo‐electron diffraction and tomography, serial‐femtosecond crystallography, and X‐ray free electron laser studies [XFEL, Fig. 8(a)], each of which require definition of new PDBx/mmCIF data items and enhancements of the wwPDB OneDep system.

In parallel, structural biology is undergoing a dramatic transformation as our *Data Depositors* begin using Integrative or Hybrid Methods (I/HM) for studying ever more complex biological systems^63^ [Fig. 8(b)]. In 2014, the wwPDB partnership took the lead in this arena by assembling a broadly representative wwPDB I/HM Task Force,^64^ now led by Helen M. Berman, Andrej Sali, Torsten Schwede, and Jill Trewhella. Building on PDBx/mmCIF, this group developed an I/HM data dictionary and constructed a prototype system for depositing I/HM structures (PDB‐Dev).^65^ Considerable challenges now lie ahead for the RCSB PDB, our wwPDB partners, and members of the nascent federation of structure data resources that will interoperate with the PDB as we develop tools for deposition, biocuration, validation, archiving, and dissemination of I/HM structures, experimental data, and metadata.

With these new experimental tools and approaches will come ever more complex three‐dimensional structures, requiring new data representations, new structure visualization tools, and integration of structure data with new biological data resources. RCSB PDB software developers are working actively in this arena to build the tools necessary to support improve data delivery and visualization and meet the evolving needs of our *Data Consumers* worldwide.

## Postscript

Research projects are only truly complete when the results have been published and the data necessary to reproduce and build on the work are made available to all. The RCSB PDB and other wwPDB partners are committed to ensuring that experimental structural biology data in the PDB are validated, expertly biocurated, securely stored, and freely disseminated without limitations on use by researchers, educators, students, and the curious public around world in perpetuity.
